# The Correlation between Enjoying Fictional Narratives and Empathy in Japanese Hikikomori

**DOI:** 10.12688/f1000research.55398.2

**Published:** 2022-01-21

**Authors:** Francesco Panto, Tamaki Saito, Nobuaki Morita, Yasukazu Ogai

**Affiliations:** 1 Social Psychiatry and Mental Health, University of Tsukuba , Faculty of Medicine, Tsukuba, Japan, 3058577, Japan

**Keywords:** Hikikomori, Social withdrawal, Emotional transportation, Empathy, narrative therapy

## Abstract

**Background: **Hikikomori is a Japanese social withdrawal phenomenon which, in recent years, is spreading in western developed countries as well. Spending a lot of time secluded indoors, watching and playing with fictional narratives may be relatively common for Hikikomori people and may represent a protective factor for their psychological well-being.

** Method:** We evaluated the role of enjoying fictional narratives on empathy, relaxation, depression, and anxiety in people with Hikikomori experience, in relation to their daily consumption of fictional narratives and their emotional transportation toward fictional narratives. Hikikomori from one psychiatric clinic and three different support facilities were enrolled in this study. Multidimensional empathy scale, CES-D, STAI questionnaire, and relaxation inventory self-report scale were used as outcome measures.

** Results:** We found a significant correlation between empathy and emotional transportation toward fictional narratives and between relaxation during watching and reading fictional narratives and consumption frequency of fictional narratives.  We failed, however, to find any significant correlation with depression and anxiety.

**Conclusions:** These findings suggest a possible correlation between fiction and empathy/relaxation response; however, any causal relationship is not proven, consequently we deem that further investigations with a larger sample size are required for a better understanding.

## Introduction

Hikikomori could be defined as a socio-medical condition originally manifested in Japan during the end of the twentieth century. The core feature of Hikikomori is considered to be a withdrawal with various degrees from social life. Hikikomori people spend most of the day confined indoors. Avoidance of social situations and social relationships, and functional impairment of social interactions are usually present. According to the Japanese Ministry of Health, Labor and Welfare ( Saito, 2010), Hikikomori is defined as “a situation where a person without psychosis is withdrawn into his/her home for more than six months and does not participate in society such as attending school and/or work”. Lifetime prevalence of Hikikomori in Japan is said to be 1.2% ( Koyama *et al*., 2010). A Japanese cabinet report has estimated the actual number of Hikikomori in Japan as around 540,000 ( Tajan *et al*., 2017), yet this estimation varies a lot among experts. For the general public staying indoors continuously is classically considered as a necessary feature of being Hikikomori. However, according to Japanese experts ( Saito, 1998), the Hikikomori condition refers to a withdrawal from social situations, activity, and relationships with others without necessarily being physically restrained indoors. In Japan a large number of Hikikomori are still considered as such even if they visit hospitals, psychiatric clinics, employment support facilities, or even when they have an employment ( Otaya, 2015). Originally considered as a cultural-bound syndrome profoundly embedded with cultural peculiarities of Japanese society, nowadays Hikikomori is reported in different economically developed countries ( Lee *et al*., 2015).

Kato *et al*. (2011) found that among 124 mental health professionals from eight countries, Hikikomori-like phenomena were reported, furthermore differences among countries were not significant, suggesting that cultural etiology could not be the only cause behind the phenomenon ( Kato *et al*., 2011). Hikikomori case studies have been conducted from several countries outside Japan including, Spain, Oman, the United States, Canada, Italy, the United Kingdom, France, Taiwan, and South Korea ( Lee *et al*., 2015; Malagón-Amor *et al*., 2014; Sakamoto *et al*., 2005). In a study including 2,313 Japanese people, Hihara *et al*. (2021) found that in young adults prosocial behaviors positively predicted positive identity elements, on the contrary Hikikomori symptoms and suicidal ideation positively predicted negative identity elements. These findings were significant only for socioculturally relevant indicators. This could suggest the role of sociocultural elements in Hikikomori phenomenon.

For the etiology of Hikikomori there are variegated and yet contradicting points of views from experts. Hikikomori is not classically considered a psychiatric diagnosis. Some cases of Hikikomori meet the criteria of existing disorders in the DSM V (or ICD- 10), yet a consistent subset of cases does not meet the criteria of any existing psychiatric disorders, motivating some researchers to propose Hikikomori as a cultural-bound syndrome ( Teo & Gaw, 2010). For example, Imai et al. found that anxiety scores were significantly higher in Hikikomori patients. ( Imai *et al*., 2021). Among western sociologists there are researchers who suggest that Hikikomori do not have inherent psychopathology, consequently, it can represent a transient phenomenon caused only by social factors ( Furlong, 2008). Even if we fail to ascribe the Hikikomori phenomenon to a precise and univocal DSM V or ICD-10 diagnosis, it is still undeniable that people suffering from Hikikomori battle with a variegated cluster of psychiatric symptoms that results in impairing their daily activities. According to research by Kondo ( Kondo *et al*., 2013) in about 148 Hikikomori patients out of a 337 total who presented to medical institutions to be treated, a medical diagnosis was made according to DSM-IV-TR criteria. Schizophrenia, anxiety disorder, mood disorder, adjustment disorder, personality disorder (including six with avoidant personality disorder, schizoid personality disorder, obsessive-compulsive personality disorder) were commonly found ( Suwa & Suzuki, 2013). Even if the Hikikomori phenomenon is not a disease “per se”, a prolonged social withdrawal could trigger psychiatric symptoms. Hikikomori is generally described as a non-psychotic phenomenon. Even if psychiatric comorbidities may exist. A small but significant proportion of Hikikomori patients don't meet the criteria for any current diagnostic category. Tan, Lee & Kato (2021) suggested that a standardized diagnostic measure such as the 25-item Hikikomori Questionnaire should be used to assess this condition. Saito (2009) described the Hikikomori personality as characterized by low self-esteem and a tendency to feel anxious and nervous in front of other people.

In regard to the correlation between Hikikomori and internet addiction, there is not univocal consensus, as many researchers seem to point to internet use and online communication as a predisposing factor leading to social withdrawal ( Taylor, 2006). Internet addiction and Hikikomori could also function as a comorbidity of the other. According to one study conducted In South Korea, 56% of Hikikomori are at risk of internet addiction, using the internet as their only way of communicating with the world ( Lee *et al*., 2013). On the other hand, it has been suggested that internet usage, being the preferred communication and social tool used by Hikikomori, could be paradoxically beneficial for a Hikikomori’s quality of life ( Chan & Lo, 2014). It could be a potential way to interact with medical professionals or even represent a means to attend school. In this regard it is worth mentioning that in Japan a rising popular online school, called Nko (Nkoutougakkou) allows futoko (students with school refusal) and Hikikomori individuals to attend school directly from their rooms, becoming a precious tool that links them to a real community. In Japan, fictional narrative (mostly in the form of Anime and Manga) consumption behavior is classically related to the term Otaku. The term Otaku refers to “people who are interested in a specific genre or object, and are extraordinarily knowledgeable about it, but are lacking in social common sense” ( Kam, 2013). Generally speaking, in Japan, the word Otaku is used to speak about people who have a high consumption of anime or manga productions and related goods. The relationships between Hikikomori and Otaku is not quite clear yet. Some Japanese experts have suggested that Hikikomori individuals often refuse to be labeled as Otaku for the social stigma linked to the word ( Saito, 2009) Nevertheless, Saito (2009) also states that young otaku(s) in Japan tend to be bullied at school due to the presence of a “school caste system” that judges otaku students incapable of meaningful communication with peers. So, in the end younger otaku(s) tend to become Hikikomori in their adult life, developing an inferiority complex toward others. For these reasons we can assume that a part of Hikikomori population enjoys fictional narratives in their daily life. Asking Hikikomori individuals if they habitually use fictional narratives in their daily life and assessing the psychological effects of this activity, with this study we want to make an attempt in understanding this relationship. If a beneficial relationship is established, in the future we could use a form of remote narrative therapy to help Hikikomori. This therapy would be based on an activity which they already enjoy on a daily basis. This new narrative therapy approach could represent a tool of intervention in a population reluctant to get in touch with mental health facilities.

the authors are thinking about a new form of intervention using an original game. Some worth to mention similar programs which showed efficacy are Play Therapy (sense of well-being and psychological capital), ( Chan, 2019), and SPARX (a new computerized cognitive behavioral therapy intervention) which showed efficacy in reducing depressive symptoms in adolescents ( Merry *et al*., 2012).

### Fictional narratives and their psychological effects

“Fictional narratives” is a term used to classify activities that comprise enjoying a production which does not include actual factual information, like watching movies or anime, reading novels, or playing video games. They are fictional because they are not based on real facts or stories (or at least not entirely). ( Busselle & Bilandzic, 2008). Enjoying fictional narratives is something everybody has experienced at least once in their lifetime and it has been suggested to benefit the personal insight of individuals, giving opportunities for self- discovery and self-insight ( Green & Brock, 2002; Oatley, 1999; Oatley, 2002; Pelowski & Akiba, 2011). The mechanism through which this is deemed to be possible has been postulated by Green & Brock in their transportation-imagery model or emotional transportation theory ( Green & Brock, 2002; Green & Donahue, 2009), and is thought to be possible through emotional transportation. Emotional transportation is defined as being emotionally involved with fictional characters of a fictional story. The spectator of a fictional narrative production, if certain personal and environmental preconditions are present, will fall into a state of emotional detachment and be transported in the world of the story ( Van Laer *et al*., 2014). This process, described in the transportation theory, may be mediated, according to Van Lear, by the empathy the spectator grows toward the story characters and the vivid imagination of the plot occurring in the mind of the spectator ( Coplan, 2004). The process implicated in the transportation imagery model by Green & Brock could lead to different effects on the psyche of the spectator. One could be the enhancing of empathy toward other people. It has been suggested that people who enjoy a large quantity of fictional narrative productions become more empathetic towards other people ( Mar *et al*., 2006). One possible interpretation that has been raised is that, for the mind of the spectator, fiction functions as a simulation of human and social interactions, therefore narrative may function as an indirect learning experience ( Argo *et al*., 2008). In contrast, some researchers suggest that a possible explanation for fiction positively associated with empathy is that more empathetic people tend to enjoy more fiction in the first place ( Bal & Veltkamp, 2013). The debate whether fiction influences empathy or not is still ongoing, but Bal & Veltkamp (2013) with an experimental approach demonstrated an undeniable influence of fiction compared to non-fiction to the empathy of the individual, but only when low or high emotional transportation into the specific story enjoyed occurred ( Busselle & Bilandzic, 2009). The mediating role of emotional transportation in influencing empathy skills may be the main candidate in predicting empathy ( Busselle & Bilandzic, 2009; Gerrig, 1993). Aside from emotional influence, the narrative transportation model predicts that narratives can induce a cognitive persuasion on the spectator, which is strong and long-lasting ( Coplan, 2004). In other words, fictional narratives may trigger a cognitive transformation experience ( Green & Brock, 2000; Phillips & Mcquarrie, 2010). Further investigations on this field lead to the distinction between analytical and narrative persuasion ( Petty & Cacioppo, 1986). Compared to analytical persuasion of factual stories, narrative persuasion is not overtly persuasive, is not inherently critical and does not involve scrutiny, characteristics that allow persuasion to be more effective and long-lasting ( Appel & Richter, 2007; Locke, 1987; Slater, 2002). The narrative persuasion evoked by fictional narratives could be linked to the Social Learning theory of Bandura ( Evans *et al*., 1970). In this case, observation and subsequent behavioral imitation stems not from people in actual society but from fictional narrative characters.

Fictional narratives could be used as a form of narrative therapy. In this study we chose to examine also the relationship with depression and anxiety because there are evidences that narrative therapy is correlated to an improvement in these dimensions. For instance, in a randomized trial measured the effects of Narrative therapy for depression and anxiety among children with imprisoned parents, the findings showed an improvement in depression and anxiety for the intervention group. ( Jalali *et al*., 2019). Another study assessed the the effectiveness of group narrative therapy on depression, quality of life (QoL), and anxiety among people with amphetamine addiction showing a statistically significant reduction in depression and anxiety scores ( Foroushani & Foruzandeh, 2015). Other studies showed similar effects on depression and anxiety using narrative therapy. ( Lopes *et al*., 2014; Naziri *et al*., 2010)

### Hikikomori and fictional narratives and study rationale

According to a fact-finding investigation about Hikikomori conducted by the Japanese Ministry of Health, Labour and Welfare, in 2010, concerning activities engaged in indoors, Hikikomori individuals engaged more in the reading of books, playing video-games and using internet compared to healthy individuals ( Japanese Ministry of Health, Labour and Welfare, 2010). Yet, actually there is no data regarding Hikikomori engaging in fictional narratives in their daily lives more than healthy individuals. The rationale of this investigation is based on the hypothesis that Hikikomori individuals that engage more often in fictional narratives, or who are more easily emotionally transported to a story tend to be more empathetic at the same level of social impairment. We want also to verify if Hikikomori individuals, who engage in fictional narratives more often or have a higher level of emotional transportation, have accordingly lower levels of the anxiety and depression associated with their condition. According to previous researches, there are few but significant findings of the relationship between empathy and fictional narrative consumption. However, to the authors’ knowledge, there are no studies that try to estimate if the consumption of fictional narratives may or may not enact as a protective factor against depression or anxiety in socially impaired individuals. If a beneficial correlation is established it could be worth trying to develop a method of intervention using fictional narratives (anime, games) to support Japanese socially impaired individuals.

## Methods

### Participants

In this study, we enrolled only Hikikomori who already got in touch with medical facilities (psychiatric clinics) or non-profit support facilities providing Hikikomori individuals - self-help groups or employment support. Being difficult to get in touch with them, we excluded actively secluded Hikikomori individuals. For this reason, Hikikomori enrolled in this study are considered “people who experienced Hikikomori phenomenon” and not people who were actively secluded. Inclusion criteria were (a) belonging to a support facility for Hikikomori or be treated for Hikikomori as an outpatient or a daycare patient in a psychiatric clinic or hospital. (b) being over 18 years old. (c) being Japanese and (d) having experienced Hikikomori condition for over six months in the past. Participants were excluded if they presented (a) other psychiatric symptoms aside from social seclusion and impairment (b) psychotic symptoms.

### Procedures

The study received ethical approval from the Bio-Ethical Committee of Tsukuba University (ethical approval number 1247). We conducted a cross- sectional questionnaire survey from January 2018 to December 2018 in four different facilities. Three of them were non- profit support organizations and one was a psychiatric clinic (in this case we recruited patients in the daycare facility and in the outpatient care as well), all located in the Kanto area, Japan. The recruitment procedure consisted of a periodic explanation by the authors of the research purpose and research protocol to Hikikomori individuals attending the support facilities and psychiatric clinic. Hikikomori individuals interested in the questionnaire were instructed to contact the facility staff afterward. After an evaluation of the patient eligibility by the authors the questionnaires were distributed to participants. The participants could either answer the questionnaire directly right after the distribution or they could bring it home and give it back afterward to the facilities staff. After data collection, we provided a participation incentive (a 500yen prepaid card) to all participants who completed the questionnaire.

## Measures

### Outcomes measures

**The Center for Epidemiologic Studies Depression Scale, Japanese version (CES-D).** The CES-D is considered one of the gold-standard measures for depression. It a brief self-report 20-item measure that asks participants to rate how often over the past week they experienced symptoms associated with depression ( Lewinsohn *et al*., 1997). Response options range from 0 to 3 for each item (0 = Rarely or None of the Time, 1=Some or Little of the Time, 2=Moderately or Much of the time, 3 = Most or Almost All the Time). Scores range from 0 to 60. The cutoff score for clinical depression is 16 or greater. We decided to use the CES-D to assess any difference in depressive symptoms between Hikikomori individuals with a high and low rate of fictional narrative consumption and with high and low scores in emotional transportation. For this study we defined high emotional transportation as higher scores on the Narrative Transportation Scale compared to lower score in the same scale (low narrative transportation).

**The State-Trait Anxiety Inventory ( Spielberger, 1983).** The State-Trait Anxiety Inventory (STAI) is a scale used to measure trait anxiety. It is generally used in clinical settings from physicians and clinical psychologists to diagnose anxiety. In this study, we implemented the 20 items version. All items are rated on a four-point-scale (e.g., from “Almost Never” to “Almost Always”). Higher scores indicate greater anxiety. This scale is considered one of the most valid and reliable instruments to assess clinical anxiety ( Suzuki & Kino, 2008).

We decided to measure trait anxiety (vs. state) because we supposed a possible relationship between a tendency to be immersed into narratives stories and fictional consumption and a lower anxiety trait. In other words, we expect that a habitual higher fictional consumption and transportation could predict a long-term lower anxiety trait.

**Multidimensional Empathy Scale.** Multidimensional Empathy Scale (MES)is a Japanese 24-item self-report measure of 5 dimensions of empathy, for distinctively assessing self/other- orientation of either cognitive or emotional components. The five dimensions are 1) Other-Oriented Emotional Reactivity, 2) Self-Oriented Emotional Reactivity, 3) Emotional Susceptibility, 4) Perspective Taking, and 5) Fantasy. (the internal consistency indexes for every sub scale were.71 .60 .78 .69 .70). A series of validation studies were made by authors to test the validity of the tool and each of the five sub-scales demonstrated a predictable pattern of relationships with existing scales like Interpersonal reactivity Index IRI or Questionnaire Measure of Emotional empathy QMEE ( Mehrabian & Epstein, 1972).

**Short-form self-report measure to assess relaxation effect (S-MARE), ( Sakakibara *et al*., 2014).** S-MARE is a relatively new self-report tool to assess relaxation effect based on the Relaxation Inventory ( Crist *et al*., 1989). The item consists of three sub scales (a) physiological tension (b) psychological relaxation (c) anxiety. For each sub scale the Cronbach’s coefficient was .93, .94 and .85. S-Mare scores were significantly correlated with the Emotional Relaxation Scale ( Tokuda, 2011) (r=.446) and with State Anxiety (r= -.531) (N=172). The validity tests suggested that S-MARE has reliability and validity when correlating with cardiac parasympathetic tone, which is the physiological reaction to a relaxation stimulus. This suggests that S-MARE is a valid measure when measuring relaxation effects. Normally the assessment of relaxation effect occurs after the exposure to a relaxation stimulus evoked by a relaxation technique. For this study, we ask the participants to recall the relaxation state they usually experience while they are enjoying fictional narratives and to answer accordingly. We used S-MARE in a modified manner, for this reason the scores for this instrument may be affected by recall bias.

### Predictor measures

**Narrative Transportation Scale (NTS- J).** Japanese version of the scale ( Osanai & Kusumi, 2016). NTS-J consists of 12 items and is a seven-point scale (from 1=not fitting at all to 7=fitting perfectly). Transportation into narratives is considered a mechanism by which narratives can affect beliefs. The elements included in the transportation experience are imagery, affect, and attentional focus. The Narrative Transportation Scale (NTS-J) was developed firstly by Green & Brock (2000). A shorter version of the same scale was developed by Appel *et al*. (2015). According to Green and Brock transportation is associated with story-consistent beliefs, meaning that highly transported participants had beliefs more consistent with the story and showed a more positive evaluation of characters. Although causality is not established, the persuasion effects of narratives are broadly demonstrated, meaning that highly transported individuals changed their real-world beliefs in response to experiences in a fictional world ( Osanai & Kusumi, 2016). Japanese confirmatory studies showed the reliability of the scale and the correlation in measuring imaginative involvement and literary response. ( Bal *et al* ., 2011) Osanai & Kusumi's scale required the narrative task "Kin no Wa" and "Chiyogami no Haru" to the respondents. For this study, we instructed the participants not to limit the response to a single fictional production but to answer accordingly to their habitual emotional response to fictional narratives.

This modified use of NTS-J is supported by literature that claims a difference in individuals based on their transportability into narrative worlds ( Gnambs *et al*., 2014). Transportation has been linked to individual differences in trait empathy and trait absorption ( Green & Donahue, 2009). Some authors describe transportation as an immersive tendency positively related to the Big Five dimensions of openness, neuroticism, and extraversion ( Weibel *et al*., 2010). This trait transportability may define individual differences in becoming transported into narrative stories (e.g., Busselle & Bilandzic, 2008; Mazzocco *et al*., 2010).

### Consumption of fictional narratives

In this study, we decided to set as a predictor measure “habitual consumption of fictional narratives”. We asked if participants had or did not have habitual consumption of fictional narrative productions (movies, anime, manga, novels, drama, or games). We asked if they enjoy fictional narratives “habitually”, “from time to time “and “nothing at all”. For the data analysis, we decided to set two groups. One that comprised only participants with habitual consumption of fictional narratives, the other which included participants who enjoy fictional narratives from time to times and participants who don’t enjoy fictional narratives completely.

### Descriptive measures

**Hikikomori status evaluation.** In order to make an evaluation about the participants’ Hikikomori status we employed a few questions extracted from the “Hikikomori status evaluation chart” implemented by Japan's Ministry of Health, Labour and Welfare, currently used by Japanese psychiatrists ( Saito, 2010). Specifically, we asked about the age of onset of Hikikomori phenomenon, the perceived reason linked to the Hikikomori behavior, the number of friends, the experience of being bullying or bullying perpetrating behavior, and the use of internet and TV in daily life. We used a consensus evaluation measure of Hikikomori issued by the Japanese Cabinet Office after the 2016 Survey of Acute Social Withdrawal ( Tajan *et al*., 2017).

## Data analysis

To test the Hypothesis that Hikikomori participants with high fictional narrative consumption rates and Hikikomori participants with low fictional narrative consumption rates were associated with the statistically significantly different mean of scores in the outcomes variables, an independent samples t-test was performed. To test the hypothesis that higher narrative transportation skills or high fictional narrative consumption rates were positively correlated with higher empathy and relaxation, and negatively correlated with anxiety or depression we performed a Pearson correlation test. Afterward, for statistically significant results, multiple regression analysis was performed. The level of statistical significance was set at *p*<.05. All data analysis was performed using SPSS v. 25 (IBM SPSS Statistics, RRID:SCR_019096).

## Results

**Figure 1.**
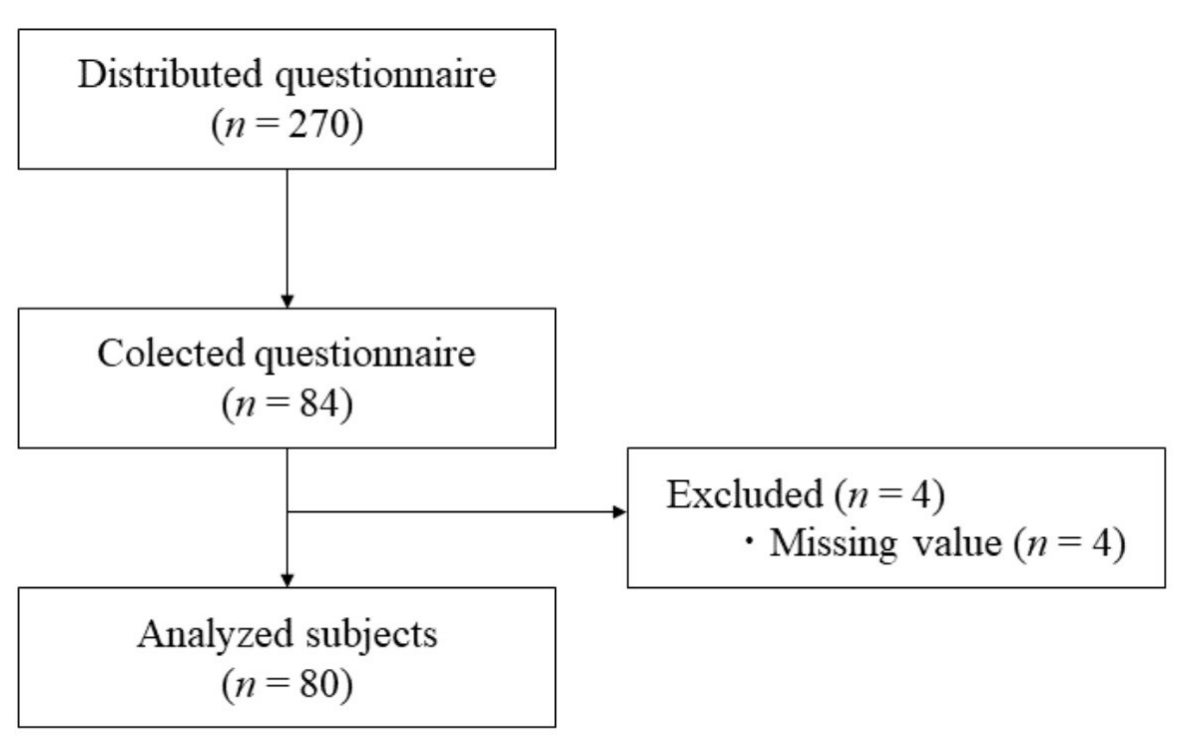
Flow chart of data collecting process.

A total of 270 questionnaires were distributed, 84 responses were collected, 4 were excluded from the analysis due to extensive missing values. The remaining 80 were suitable for the analysis. To deal with the remaining amount of missing values, an Expectation Maximization algorithm was used for missing data analysis. Overall, less than 5% of data were missing from the dataset(n=80). A non-significant Little’s MCAR test, χ2(281) = 309.403, p = .11, revealed that the data were missing completely at random. Missing data were imputed using Missing Values Analysis within SPSS 25. A flow chart of the data collection process is shown in Figure 1. The average age (years) of participants (n=80) was 36 (SD 8.6). The period in which they started Hikikomori behavior, the reason they started to actively recluse themselves, the presence or absence of bullying experience, and the number of friends are reported in Table 1. Regarding fictional narrative consumption behavior, the participants’ tendencies are shown in Table 2. We also asked the main reason why they enjoy fictional narratives when they do and the positive emotions elicited by consumption behavior as well as if they experience a decrease in negative emotions during consumption behavior or not, Table 2. To test the hypothesis of a correlation between fiction-consumption-related behavior (emotional transportation and fiction consumption rate) and the dependent variables, a Pearson correlation analysis was conducted. There was a positive correlation between emotional transportation and empathy, r = .498, p = < .001, n = 80. Also, there was a positive correlation between fiction consumption rate and relaxation, r = .280, p = < .005, n = 80, Table 3. An independent-samples t-test was conducted to compare relaxation, empathy, depression, and anxiety levels in Hikikomori with habitual consumption of fictional narratives and Hikikomori without habitual consumption of fictional narratives. There was a significant difference in the scores for habitual consumption (M=53.0, SD=12.4) and non-habitual consumption (M=46.3, SD=8.7) only for relaxation, Table 4. When we compared the level of relaxation between Hikikomori with habitual consumption and no habitual consumption (from time to time group and nothing at all group) of 3 groups, there was a statistically significant difference between groups as determined by one-way ANOVA (F (2,77) = 3.534, p = .034), Table 5. A multiple regression was carried out to investigate whether emotional transportation and fiction consumption rate could significantly predict participants ‘empathy. The results of the regression indicated that the model explained 23.2% of the variance and that the model was a significant predictor of empathy, F (2,77) = 12.94, p<.01. While emotional transportation contributed significantly to the model (B = .551, p=.000), fiction consumption rate did not (B = -1.41, p=.539), Table 6. For relaxation, the results of the regression indicated that the model explained 5.5% of the variance and that the model was a significant predictor of relaxation, F (2,77) = 3.30, p = 0.042. While fiction consumption rate contributed significantly to the model (B = 6.61, p=0.14), emotional transportation rate did not (B = -0.26, p=.84), Table 6.

**Table 1.** Descriptive statistics of participants.

	Total sample	%
Sex		
Male	60	75
Female	20	25
Age (Years)		
18–30	22	27.5
31–39	31	38.8
40–49	21	26.2
50 or over	6	7.5
Starting period of Hikikomori behavior		
From elementary to senior high school	41	53.2
From university to employment	36	44.2
Reason of Hikikomori behavior		
Failure experience at school	9	11.3
Failure experience at work	6	7.5
Change in school environment	10	12.5
Change in work environment	5	6.3
Friction in family relationships	23	28.7
Friction in other relationship	9	11.3
Other	16	12.6
Experience of bullying or being bulled		
Experience of being bulled	48	62.7
Experience of bullying	25	32.6
Number of friends		
No one	17	21.8
At least one friend	6	7.7
At least two friends	55	70.5

**Table 2.** Narrative consumption tendencies in participants.

	Total sample	%
Fiction consumption		
Habitual consumption	46	57.5
Occasional consumption	25	21.3
Absence of consumption	9	11.3
Reasons for fiction consumption behavior		
Killing time		9.8
Enjoying the contents		7.0
Emotional transportation		15.4
Feeling the mood supported		15.4
Escapism from reality		12.9
Feeling uplifted		12.9
Refreshing		7.8
Other (loving for the character, to learn about life, to foster creativity, custom)		18.8
Positive emotions elicited by fiction narrative		
Not affected		24.4
Feeling more amused		9.4
Feeling more motivated		24.8
Feeling more hopeful		22.6
Feeling more encouraged		18.8
Negative emotions affected by fiction narrative		
I forget almost entirely my negative mood	9	11.3
I don’t forget my negative mood but I feel better	59	73.8
I retain entirely my negative	11	13.8

**Table 3.** Correlation between outcome variables and predictor variables.

	1	2	3	4	5	6
1. Emotional transportation	-	.297 **	.062	.498 **	0.37	-.074
2. Fiction consumption	.297 **	-	.280 *	0.90	-.082	-.214
3. Relaxation	.062	.280 *	-	.097	-.598 **	-.510 **
4. Empathy	.498 **	.090	.097	-	.168	.183
5. Depression	0.37	-.082	-.598 **	.168	-	.786 **
6. Anxiety	-.074	-.214	-.510 **	.183	.786 **	-

* *p*< .05. ** *p*< .01

**Table 4.** T-test results comparing habitual and non-habitual consumption of fictional narratives on outcomes.

	Habitual consumption		Non-habitual consumption		
	*M*	*SD*	*M*	*SD*	*t* test
Empathy	83.0	11.0	81.0	11.1	-.79
Relaxation	53.0	12.4	46.3	8.7	-2.6 *
Depression	49.5	9.4	51.2	11.4	-.72
Anxiety	58.4	7.9	62.1	9.0	1.9

* *p*< .05. ** *p*< .01

**Table 5.** one way ANOVA for relaxation between fictional narrative consumption of 3 groups.

	Sum df of squares	Mean square	*F*	Sig.
Between groups	869.887	434	3.53	.034
Within groups	9476.398	123		
Total	10346.285			

**Table 6.** Multiple regression for emotional consumption and fictional consumption predicting empathy and relaxation.

	Predictors	(B)	*SE*(B)	*β*	Sig.( *p*)	*t*
empathy	Emotional Transportation	.551	.110	.517	.000	5.0
	Fictional consumption	-1.41	.2.29	-.064	.539	-.61
relaxation	Emotional transportation	-0.26	.127	-0.23	.840	-.20
	Fictional consumption	6.61	2.63	.287	.014	2.5

*Note.* Empathy *R* ^2^= .232. Relaxation *R* ^2^= .05

## Discussion

This study wanted to explore the connection between empathy relaxation, depression, anxiety, and fictional narrative consumption behavior by Hikikomori Individuals in Japan in order to introduce a new form of narrative therapy for Hikikomori individuals. Due to the lack of data regarding firstly, the influence of habitual consumption of fictional narratives on empathy relaxation, depression anxiety in the general population, moreover in Hikikomori individuals, the outcome setting was mainly exploratory. According to the results pertinent to the outcomes, t-test results have shown a statistically significant difference only for relaxation response in regard to fictional narrative consumption behavior. This could suggest that while consuming fictional narratives, Hikikomori individuals with habitual fictional narratives consumption behavior tend to have a higher relaxation response. For the correlation analysis and the multiple regression analysis conducted using transportation as a continuous variable predicting empathy, we obtained a pattern of results consistent with previous research that showed a correlation between emotional transportation and empathy. However, considering the positive correlation between depression and trait anxiety, the results of the regression analyses may be affected by multi-collinearity.

Although correlation doesn’t mean causality, in previous research measuring the effects of emotional transportation on fiction and nonfiction readers, it was shown that empathy was influenced only for fiction readers and not for nonfiction, suggesting the validity of emotional transportation theory ( Busselle & Bilandzic, 2009). According to the transportation theory and its further developments, in order to change as a consequence of enjoying fictional narratives, the individual has to be emotionally transported, otherwise distraction from the story and consequently, disengagement from the story will occur ( Goldstein, 2009; Mar *et al.*, 2009). Goldstein explains further the relationship existing between fiction and empathy. Compared to factual information, fiction is mainly oriented at eliciting emotions. For the spectators, while they are engaging in the characters emotions, the realistic aspect of the story loses its importance and does not act as a hindrance to experiencing characters emotions. On the contrary fictionality of the story could constitute a safe arena for the spectator, protecting from defense mechanisms while allowing the spectator to experience both his and the characters emotions ( Eisenberg & Miller, 1987; Goldstein, 2009; Grant, 2008; Grant & Berry, 2011; Stocks *et al*., 2008). Finding a way to enhance empathy toward others could be meaningful from a psychological and social point of view especially for socially impaired individuals like Hikikomori. By promoting social behavior, empathy is considered to be a pro-social factor. Empathy is also considered to be related to individual creativity and cooperative behavior in the workplace ( Dumtrache, 2014; Stocks *et al*., 2008). If it is true that empathy fosters social behavior, the other way around is also possible, that social contact is essential to maintain empathy skills. According to the social support theory ( O’Reilly, 1988), social networks providing emotional concern (love, empathy, liking), instrumental aid, information, and appraisal, are regarded as a basic requirement for existence ( Langford *et al*., 1997). Hikikomori lacking healthy contacts with social networks could exist in a situation in which empathy skills are not promoted or maintained by exchanges with other individuals. In this light, if in future studies causality will be proven, the enhancing of empathy related to consumption of fictional narratives could be meaningful in helping them not to weaken interpersonal skills during social isolation. Aside from empathy, T-test, Pearson correlation and Multi-regression analysis confirmed a relation between the habitual consumption of fictional narratives and relaxation effect. The relaxation effect measured in this study was linked to the physiological and psychological relaxation experienced during consumption of fictional narratives, as we instructed the participants to refer to their state during fictional narrative experiences. According to previous studies, the relaxation effect is correlated with emotional relaxation, physiological tension, and state anxiety. Hikikomori individuals who habitually enjoy fictional narratives or have higher emotional transportation skills tend to be more relaxed while watching a movie or reading a book. Even if the causality of this relationship is not proven and no correlation between predictor variables and anxiety and depression was found in this study, we can surely give some possible interpretations of the relaxation effect. It could be that people with high emotional transportation or fictional narrative consumption habits, have less risk of becoming detached, are more able to immerse themselves in the story and this could act as a protective factor against physiological and emotional tension. For Hikikomori individuals this could be particularly meaningful, given that the comorbidity with anxiety is very common. In this regard, we assessed anxiety with STAI in our study, and we found a mean score for anxiety that was relatively high and above the clinical threshold. The effect of fictional narratives on anxiety symptoms and emotional tension, even if the literature is quite limited, is not an unheard-of topic. In 2014 a study tested the effects of cinema therapy on diminishing anxiety in young people. Compared to a control group, participants who took cinema therapy sessions showed a lower score on the Hamilton anxiety rating scale. This suggests that Cinema Therapy could have a role in decreasing anxiety in individuals with high levels of stress and anxiety ( Dumtrache, 2014). Furthermore, another study examined the effects of watching a movie on a family member’s anxiety level during their relatives’ surgery, assessing anxiety with STAI. It was found that after watching a movie the level of anxiety was significantly reduced in people already in an anxious state due to their relative’s surgery ( Mojdeh *et al*., 2013). This suggested a possible protective role of fictional narratives towards anxiety and emotional tension ( Fatahi *et al*., 2021). Johnson (2012) conducted a study demonstrating that engaging in a fictional story fosters not only empathy but also prosocial behavior. Dunbar *et al*. (2016) proved that emotional arousal when watching drama increases pain thresholds and social bonding. These pro-social behavioral effects could be extremely useful for socially impaired Hikikomori(s). According to squared correlations, emotional transportation shared 25.0 % while fiction consumption rate and relaxation shared 9.0 %of the variance. Other possible responsible factors may be investigated in the future. In particular a study evaluating effectiveness of play therapy (online -game program) showed improving in empowerment and psychological capital ( Chan, 2019). Play therapy could be considered a form of fictional narrative, so it could be worth to investigate in the future stress coping related measures. However, another possible explanation for socially impaired people enjoying fiction narratives, could be that people with more depression and anxiety symptoms will interpret various events negatively and ruminate them repeatedly. In other words, lower depression and anxiety or higher relaxation predicting a tendency in enjoying Fictional Narratives is a possible convincing hypothesis. Also, Nonaka & Sakai (2021) found that stress coping skills, self-compassion, and psychological stress were associated with Hikikomori. The use of instruments which support these dimensions were associated with a decrease in Hikikomori related behaviors ( Nonaka & Sakai, 2021). Hamasaki *et al.* (2021) evaluated environmental factors in adolescent Hikikomoris. “anxious/depressed,” “somatic complaints,” “lack of communication between parents” and “overuse of the Internet” were significant predictors of hikikomori severity. Even if the overuse of internet is considered a predictor of severity, no data exist regarding fiction Narrative consumption. In the future the authors are planning to explore the efficacy of an internet based narrative program to understand further the possible positive and supportive role of internet in the life of Hikikomori patients. Targeting with a computerized interactive program self-compassion, and other psychological stress dimensions could be useful as a tool of support for these patients.

However is worth to mention that Saito (2020) points out how Hikikomori in their middle age tend to hide their interest in entertainment because of a sense of guilt regarding recreational activities. Finally, with regard to depression and anxiety, no statistically significant result was observed, however a negative correlation between Anxiety, Depression, and Relaxation was observed. With this study, with its relatively small sample size, low coefficients results, and a cross-sectional nature, we couldn’t draw any conclusive evidence about the psychological effects of fictional narrative consumption behavior, but the data suggest a possible correlation of fictional narrative consumption behavior with relaxation and empathy.

## Study limitations

This study incorporated several limitations mainly related to the peculiarity of the target group. Firstly, recruiting Hikikomori individuals was quite a challenging task, for this reason, we had to deal with a limited sample size to test the study hypothesis. Due to the exploratory nature of the study and the lack of scientific data related to Hikikomori fictional narrative consumption behavior, the selection of questionnaire items was not sufficiently endorsed by literature. Finally, maybe due to the difficulty in answering about psychiatric symptoms like depression and anxiety, we resulted in having participants reluctant in answering the questions which curtail the sample size even further. To overcome these limitations, a research design which includes internet survey may be the ideal solution, reaching directly to secluded Hikikomori individuals and lowering the risk of failed responses to questionnaire items.

## Conclusions

This study aimed to explore the situation of empathy, relaxation, anxiety, and depression of Hikikomori individuals related to their consumption of fictional narrative productions and their emotional transportation skills. Although the relationship with anxiety and depression could not be assessed due to the limited sample size, the results obtained for empathy and relaxation are consistent with the previous research done with healthy individuals. This may suggest the possible benefits of fictional narratives on enhancing empathy and diminishing emotional tension in Hikikomori individuals. Compared to healthy people, these results could be even more meaningful for Hikikomori individuals, given that they are subject to a higher level of anxiety and impaired social behavior compared to the general population. Considering the limitations of the study and the fact that only a correlation has been proven, the results should be interpreted with caution. To test the hypothesis for emotional transportation or fictional narrative consumption behavior to be predictors of empathy, relaxation, or other psychological dimensions, future research with an interventional study design is needed for a better understanding of causality before trying to establish a new experimental approach using narrative therapy for Hikikomori individuals. The usage of narrative therapy in adolescents seeking help in Japan is not an unheard topic. Iejima (2006) showed that Narrative therapy using manga liked by the clients (Japanese adolescents ) could be useful in improving self-awareness. Wada *et al.* (2017) described Manga digital storytelling as a potential learning tool. In the future, the authors want to build a digital program which uses characters and story in a narrative approach. This could be a very accessible tool for Hikikomori to get in touch with medical facilities in a casual an easy way.

## Data availability

Repository: Uploaded to Harvard Dataverse

Panto, Francesco, 2021, "The Correlation between Enjoying Fictional Narratives and Empathy in Japanese Hikikomori", https://doi.org/10.7910/DVN/8AYPKV, Harvard Dataverse, V1, UNF:6:cvasikAbeDexFrSerq36EA== [fileUNF]

this project contains the following underlying data:

• Data file 1. (Test score results in empathy, relaxation depression, anxiety, narrative fiction consumption and other descriptive measures in 80 Hikikomori patients.)

Data are available under the terms of Harvard Dataverse “No rights reserved"

## Participant consent

Written informed consent for publication of the participants details was obtained from the participants/parents/guardian/relative of the participant.
