# Supplementary material for: The Correlation between Enjoying Fictional Narratives and Empathy in Japanese Hikikomori
Source: F1000Res. 2022 Jan 21;10:776. Originally published 2021 Aug 9. [Version 2] doi: 10.12688/f1000research.55398.2 (PMC8921688; doi:10.12688/f1000research.55398.2)
Supplement: Peer review history

# Peer review: Reviewer response for version 2

**DOI:** 10.5256/f1000research.116159.r120633

**Published:** 2022-03-14

**Authors:** Gloria Hongyee Chan

**Affiliations:** 1 Department of Social and Behavioral Sciences, City University of Hong Kong, Tat Chee Avenue, Kowloon Tong, Kowloon, Hong Kong

Given that the previous concerns have been addressed, there are no further comments.

Is the work clearly and accurately presented and does it cite the current literature?

Partly

If applicable, is the statistical analysis and its interpretation appropriate?

Partly

Are all the source data underlying the results available to ensure full reproducibility?

Yes

Is the study design appropriate and is the work technically sound?

Partly

Are the conclusions drawn adequately supported by the results?

Partly

Are sufficient details of methods and analysis provided to allow replication by others?

Partly

Reviewer Expertise:

youth in cyber space, hikikomori

I confirm that I have read this submission and believe that I have an appropriate level of expertise to confirm that it is of an acceptable scientific standard.

# Peer review: Reviewer response for version 2

**DOI:** 10.5256/f1000research.116159.r120634

**Published:** 2022-02-04

**Authors:** Ali Evren Tufan

**Affiliations:** 1 Department of Child and Adolescent Psychiatry, Faculty of Medicine, Abant Izzet Baysal University, Bolu, Turkey

The authors addressed the limitations in the previous version of the manuscript. I have no further suggestions/ comments to make.

Is the work clearly and accurately presented and does it cite the current literature?

Partly

If applicable, is the statistical analysis and its interpretation appropriate?

Partly

Are all the source data underlying the results available to ensure full reproducibility?

Yes

Is the study design appropriate and is the work technically sound?

Partly

Are the conclusions drawn adequately supported by the results?

Partly

Are sufficient details of methods and analysis provided to allow replication by others?

Partly

Reviewer Expertise:

statistics, epidemiology, child and adolescent psychiatry, developmental neuropsychiatry, biological psychiatry

I confirm that I have read this submission and believe that I have an appropriate level of expertise to confirm that it is of an acceptable scientific standard.

# Peer review: Reviewer response for version 1

**DOI:** 10.5256/f1000research.58968.r100325

**Published:** 2021-12-09

**Authors:** Gloria Hongyee Chan

**Affiliations:** 1 Department of Social and Behavioral Sciences, City University of Hong Kong, Tat Chee Avenue, Kowloon Tong, Kowloon, Hong Kong

Thank you for giving me such a great opportunity to review this article. Based on a psychiatric perspective, this article attempts to enrich the research on Hikikomori by investigating the relationship between the engagement in fictional narratives, emotional transportation, empathy, and the psychological state (level of depression and anxiety) in Hikikomori. It seeks not only to advance the study on Hikikomori but also generate practice implications, such as the use of Narrative Therapy for supporting this group of youth.

 While the potential significance of the article is highly appreciated, I would like to express my concerns as follows:

  

1. Apart from the linkage between fictional consumption and empathy, the author could provide more illustrations for studying depression and anxiety as well.
2. Given that the results of the multiple regression analysis showed that the model investigating the predictability of emotional transportation and fictional consumption rate of empathy and relaxation explained 23.2% and 5% of the variance respectively, are there possibly other factors in the model, and worth noting them as a direction for future study?
3. Narrative Therapy is suitable for deconstructing Hikikomori’s life experiences to achieve de-labeling of this group of youth and achieving empowerment of this group of youth through reconstructing their life experiences. It would be great if the author could demonstrate more the linkage of Narrative Therapy and your topic of study as practice implications.
4. As a minor comment, it would be great if taking another look at the citations (e.g., “According to one study conducted in South Korea, 56% of Hikikomori are at risk of internet addiction, using the internet as their only way of communicating with the world (Chan & Lo, 2014). On the other hand, it has been suggested that internet usage, being the preferred communication and social tool used by Hikikomori, could be paradoxically beneficial for a Hikikomori’s quality of life (Lee *et al*., 2013)” on p. 3 of the manuscript: The two references have to be swapped?)

On this account, I would suggest the author address the above concerns in order to further enhance the significance of this study.

Is the work clearly and accurately presented and does it cite the current literature?

Partly

If applicable, is the statistical analysis and its interpretation appropriate?

Partly

Are all the source data underlying the results available to ensure full reproducibility?

Yes

Is the study design appropriate and is the work technically sound?

Partly

Are the conclusions drawn adequately supported by the results?

Partly

Are sufficient details of methods and analysis provided to allow replication by others?

Partly

Reviewer Expertise:

youth in cyber space, hikikomori

I confirm that I have read this submission and believe that I have an appropriate level of expertise to confirm that it is of an acceptable scientific standard, however I have significant reservations, as outlined above.

# Response

**Published:** 2021-12-17

**Authors:** Francesco Panto

**Affiliations:** University of Tsukuba, Japan

Thank you for your kind response and your time dedicated to reading and reviewing our paper. We really appreciate your important contribution. We tried to make the specific corrections requested.

 As follows

  

1. Apart from the linkage between fictional consumption and empathy, the author could provide more illustrations for studying depression and anxiety as well.
*Thank you for your indication. We added an explanation about the reasons for choosing these measures in the paragraph about Fictional narratives.*
2. Given that the results of the multiple regression analysis showed that the model investigating the predictability of emotional transportation and fictional consumption rate of empathy and relaxation explained 23.2% and 5% of the variance respectively, are there possibly other factors in the model, and worth noting them as a direction for future study?
*Thank you for your question, I added a little paragraph in the discussion confronting the other possible measures we can use in future studies.*
3. Narrative Therapy is suitable for deconstructing Hikikomori’s life experiences to achieve de-labeling of this group of youth and achieving empowerment of this group of youth through reconstructing their life experiences. It would be great if the author could demonstrate more the linkage of Narrative Therapy and your topic of study as practice implications.
*Thank you for this important suggestion,  I added similar programs we are taking as references and their effectiveness in the introduction, as well as similar approaches using narrative therapy with fictional narratives that we can use as a model for future studies.*
4. As a minor comment, it would be great if taking another look at the citations (e.g., “According to one study conducted in South Korea, 56% of Hikikomori are at risk of internet addiction, using the internet as their only way of communicating with the world (Chan & Lo, 2014). On the other hand, it has been suggested that internet usage, being the preferred communication and social tool used by Hikikomori, could be paradoxically beneficial for a Hikikomori’s quality of life (Lee *et al*., 2013)” on p. 3 of the manuscript: The two references have to be swapped?)
*Thank you for noticing the mistake, We apologize for missing this. I corrected as you kindly suggested.*

# Peer review: Reviewer response for version 1

**DOI:** 10.5256/f1000research.58968.r95014

**Published:** 2021-10-06

**Authors:** Ali Evren Tufan

**Affiliations:** 1 Department of Child and Adolescent Psychiatry, Faculty of Medicine, Abant Izzet Baysal University, Bolu, Turkey

Firstly, I would like to thank you for providing me the opportunity to review this interesting research. The authors of this multi-center, cross-sectional, clinic-based study evaluated 80 Japanese Hikikomori with self-reports evaluating empathy (Multidimensional Empathy Scale, MES); depression (CES-D), trait anxiety (STAI), self-reported relaxation (S-MARE), and narrative transportation (NTS-J). The design was exploratory and aimed to evaluate the relationships between those constructs with an eye to developing remotely applied narrative therapy for those individuals.

 The authors report three main results;

1. Empathy levels among Hikikomori correlated with emotional transportation toward fictional narratives
2. Self-reported relaxation correlated with the frequency of consumption of such narratives
3. There were no relationships with depression and anxiety levels.

The study is novel and interesting and I would like to commend the authors, though with some reservations. Firstly, I believe the manuscript's prose may be improved for style, clarity, and flow. specifically;

- the authors may add the reference for the last sentence of the first paragraph of the introduction.
- the first sentence of the second paragraph of the introduction is a bit vague and this paragraph may be divided at the sentence outlining study aims.
- rather than "Bandura Social Learning Theory", "Social Learning Theory of Bandura" may be more appropriate.
- the sentence on excluding actively secluded Hikikomori as well as the last sentence in the procedures section should be revised for grammar.

Secondly, the methodology may be further clarified. Specifically;

- the definition of "high and low emotional transportation" for comparison of depressive scores may be added.
- the authors used the S-MARE in a modified manner (i.e. they asked the participants to recall their state after consumption of the fictional narrative) and as such, the scores for this instrument may be affected by recall bias.
- the authors also modified the use of NTS-J and reliabilities for those modifications may be reported.
- The Hikikomori status evaluation may also be improved further. The authors may add inter-rater reliabilities or whether they used consensus on status evaluations.
- the authors also reported "extensive missing values" and that they used EM algorithms for missing data analysis but they did not report their results. Those results may be added.
- the unit for age (i.e. years) may be added.
- according to squared correlations, emotional transportation shared 25.0 % of the variance while fiction consumption rate and relaxation shared 9.0 %, what other factors may be responsible and be evaluated in further studies?
- the authors reported using ANOVA for participants with habitual and not habitual consumption, n that case did they compare two or three groups? The first may be analyzed with a t-test.
- considering the positive correlations between depression and trait anxiety, the results of the regression analyses may be affected by multi-collinearity. This information may be added to the results.
- the rationale for measuring trait anxiety (vs. state) may also be further clarified.

Lastly, both the introduction and the discussion may benefit from the inclusion of some recent and related references. Suggested references may include;

- Tan *et al.* International experience of hikikomori (prolonged social withdrawal) and its relevance to psychiatric research. ^1^
-  Imai et al. The characteristics of patients with severe social withdrawal 'Hikikomori' in two community psychiatry clinics in Japan. ^2^
-  Nonaka & Sakai. Psychological Factors Associated with Social Withdrawal (Hikikomori). ^3^
-  Hihara *et al.* Positive and negative valences of identities: Longitudinal associations of identity content valences with adaptive and maladaptive functioning among Japanese young adults. ^4^
-  Hamasaki *et al.* Identifying Social Withdrawal (Hikikomori) Factors in Adolescents: Understanding the Hikikomori Spectrum. ^5^

Despite the above-mentioned limitations, the study is novel and has the potential to contribute to the literature. I commend the authors and suggest that they address those limitations which would further increase the scientific merit of the study.

Is the work clearly and accurately presented and does it cite the current literature?

Partly

If applicable, is the statistical analysis and its interpretation appropriate?

Partly

Are all the source data underlying the results available to ensure full reproducibility?

Yes

Is the study design appropriate and is the work technically sound?

Partly

Are the conclusions drawn adequately supported by the results?

Partly

Are sufficient details of methods and analysis provided to allow replication by others?

Partly

Reviewer Expertise:

statistics, epidemiology, child and adolescent psychiatry, developmental neuropsychiatry, biological psychiatry

I confirm that I have read this submission and believe that I have an appropriate level of expertise to confirm that it is of an acceptable scientific standard, however I have significant reservations, as outlined above.

## References

[1] : International experience of hikikomori (prolonged social withdrawal) and its relevance to psychiatric research. *BJPsych Int* .2021;18(2) : 10.1192/bji.2020.20 34-37 10.1192/bji.2020.20 34287404PMC8274427

[2] : The characteristics of patients with severe social withdrawal 'Hikikomori' in two community psychiatry clinics in Japan. *Asian J Psychiatr* .2021;65: 10.1016/j.ajp.2021.102833 102833 10.1016/j.ajp.2021.102833 34500393

[3] : Psychological Factors Associated with Social Withdrawal (Hikikomori). *Psychiatry Investig* .2021;18(5) : 10.30773/pi.2021.0050 463-470 10.30773/pi.2021.0050 34053211PMC8169334

[4] : Positive and negative valences of identities: Longitudinal associations of identity content valences with adaptive and maladaptive functioning among Japanese young adults. *Dev Psychopathol* .2021; 10.1017/S0954579421000043 1-15 10.1017/S0954579421000043 33766181

[5] : Identifying Social Withdrawal (Hikikomori) Factors in Adolescents: Understanding the Hikikomori Spectrum. *Child Psychiatry Hum Dev* .2021;52(5) : 10.1007/s10578-020-01064-8 808-817 10.1007/s10578-020-01064-8 32959142PMC8405474

# Response

**Published:** 2021-12-17

**Authors:** Francesco Panto

**Affiliations:** University of Tsukuba, Japan

Thank you for your detailed and accurate comments. I appreciate greatly your contribution to our research. I tried to make the specific corrections requested.

 As follows

- the authors may add the reference for the last sentence of the first paragraph of the introduction.
*I added the already listed reference which points out the presence of the Hikikomori phenomenon in other countries besides Japan.*
- the first sentence of the second paragraph of the introduction is a bit vague and this paragraph may be divided at the sentence outlining study aims.　
*I apologize if I misunderstood,  however, I divided the paragraph talking about comorbidities and the paragraph starting with the relationship with internet addiction.*
- rather than "Bandura Social Learning Theory", "Social Learning Theory of Bandura" may be more appropriate. 　
*Thank you for your accurate comment, I fixed the text with the correct terminology.*
- the sentence on excluding actively secluded Hikikomori as well as the last sentence in the procedures section should be revised for grammar.
* I apologize for the inaccurate grammar, I tried to reformulate the sentence.*
- Secondly, the methodology may be further clarified. Specifically;
  the definition of "high and low emotional transportation" for comparison of depressive scores may be added.
*I added the definition of high and low emotional transportation for this study. The definition is relative and regards the score of the scale.*
- the authors used the S-MARE in a modified manner (i.e. they asked the participants to recall their state after consumption of the fictional narrative) and as such, the scores for this instrument may be affected by recall bias.
*Thank you for pointing out this possibility, I added a sentence declaring the above.*
- the authors also modified the use of NTS-J and reliabilities for those modifications may be reported.
*I  explained the rationale for this choice as well as the pertinent literature.*
- The Hikikomori status evaluation may also be improved further. The authors may add inter-rater reliabilities or whether they used consensus on status evaluations.
* I added a statement about the consensus in the Japanese psychiatric community about the scale we used as well as the literature referring to the measures.*
- the authors also reported "extensive missing values" and that they used EM algorithms for missing data analysis but they did not report their results. Those results may be added.
*Thank you for pointing out this important detail, I added the Little’s MCAR test as well as the percentage of the missing data. However, it is necessary I can add the figure of  EM algorithms pertinent to the results for each variable.*
- the unit for age (i.e. years) may be added. 
*I added the unit.*
- according to squared correlations, emotional transportation shared 25.0 % of the variance while fiction consumption rate and relaxation shared 9.0 %, what other factors may be responsible and be evaluated in further studies?
*I tried to explain other possibilities as well as possible measures we can use in future studies.*
- the authors reported using ANOVA for participants with habitual and not habitual consumption, n that case did they compare two or three groups? The first may be analyzed with a t-test.
*I apologized for the difficulty In interpreting the text. I confirm that we used ANOVA because the comparison is between 3 groups. Specifically, 1) habitual consumption  2) no habitual consumption (from time to time group ) 3) no habitual consumption (nothing at all group)*
- considering the positive correlations between depression and trait anxiety, the results of the regression analyses may be affected by multi-collinearity. This information may be added to the results.
*I added the information, thank you for the indication*
- the rationale for measuring trait anxiety (vs. state) may also be further clarified.
* I added the explanation for this choice in the paragraph relative to the measure used for anxiety.*

 

- Lastly, both the introduction and the discussion may benefit from the inclusion of some recent and related references. Suggested references may include;
  Thank you for your kind remark, I appreciate your contribution searching for pertinent papers. I Added the suggestions as follows:

 

1. Tan *et al.* International experience of hikikomori (prolonged social withdrawal) and its relevance to psychiatric research. ⇨ *introduction*
2. Imai et al. The characteristics of patients with severe social withdrawal 'Hikikomori' in two community psychiatry clinics in Japan. ⇨ *introduction*
3.  Nonaka & Sakai. Psychological Factors Associated with Social Withdrawal (Hikikomori). ⇨ *discussion*
4.  Hihara *et al.* Positive and negative valences of identities: Longitudinal associations of identity content valences with adaptive and maladaptive functioning among Japanese young adults. ⇨ *introduction*
5.  Hamasaki *et al.* Identifying Social Withdrawal (Hikikomori) Factors in Adolescents: Understanding the Hikikomori Spectrum ⇨ *discussion*
